# Metavirome Sequencing to Evaluate Norovirus Diversity in Sewage and Related Bioaccumulated Oysters

**DOI:** 10.3389/fmicb.2019.02394

**Published:** 2019-10-17

**Authors:** Sofia Strubbia, Julien Schaeffer, Bas B. Oude Munnink, Alban Besnard, My V. T. Phan, David F. Nieuwenhuijse, Miranda de Graaf, Claudia M. E. Schapendonk, Candice Wacrenier, Matthew Cotten, Marion P. G. Koopmans, Françoise S. Le Guyader

**Affiliations:** ^1^Laboratoire de Microbiologie, LSEM-SG2M-RBE, Ifremer, Nantes, France; ^2^Department of Viroscience, Erasmus University Medical Center, Rotterdam, Netherlands

**Keywords:** norovirus, sewage, oysters, metagenomic sequencing, metavirome

## Abstract

Metagenomic sequencing is a promising method to determine the virus diversity in environmental samples such as sewage or shellfish. However, to identify the short RNA genomes of human enteric viruses among the large diversity of nucleic acids present in such complex matrices, method optimization is still needed. This work presents methodological developments focused on norovirus, a small ssRNA non-enveloped virus known as the major cause of human gastroenteritis worldwide and frequently present in human excreta and sewage. Different elution protocols were applied and Illumina MiSeq technology were used to study norovirus diversity. A double approach, agnostic deep sequencing and a capture-based approach (VirCapSeq-VERT) was used to identify norovirus in environmental samples. Family-specific viral contigs were classified and sorted by SLIM and final norovirus contigs were genotyped using the online Norovirus genotyping tool v2.0. From sewage samples, 14 norovirus genogroup I sequences were identified of which six were complete genomes. For norovirus genogroup II, nine sequences were identified and three of them comprised more than half of the genome. In oyster samples bioaccumulated with these sewage samples, only the use of an enrichment step during library preparation allowed successful identification of nine different sequences of norovirus genogroup I and four for genogroup II (>500 bp). This study demonstrates the importance of method development to increase virus recovery, and the interest of a capture-based approach to be able to identify viruses present at low concentrations.

## Introduction

Noroviruses (NoV) are small, icosahedral non-enveloped viruses, belonging to the *Caliciviridae* family, and are recognized as the predominant cause of non-bacterial human gastroenteritis worldwide. Their single strand RNA genome is short (around 7.5 k bases) and contains three open reading frames (ORFs), of which ORF1 encodes the non-structural proteins while ORF2 and ORF3 encode the major and minor capsid structural proteins (VP1 and VP2, respectively). Based on their genetic characteristics, NoV are classified into at least seven genogroups (G), from GI to GVII, which are further divided into over 30 genotypes (de Graaf et al., 2016).

Noroviruses circulate year-round but in disease surveillance distinct seasonal peaks are seen in regions with winter seasonality, reflecting dynamics of the NoV genotypes that are most commonly associated with sporadic cases and outbreaks. NoV particles released in human vomitus and stools can then be detected in sewage and may contaminate surface waters including the marine environment (Sano et al., 2016). However, a wide and increasing range of other viruses has been identified in human stools, partially associated with gastro-enteritis, but also non-mammalian viruses (Nieuwenhuijse and Koopmans, 2017). Raw sewage are rich sample types, consisting of the excreta of thousands of people, including urine, feces, and skin desquamation and all associated commensal and pathogenic bacteria, phages, and protozoa, and viruses, including viruses associated with microbiota and diet associated viruses (Nieuwenhuijse and Koopmans, 2017; Adriaenssens et al., 2018; Fumian et al., 2019). Therefore, finding NoV sequences in this complex matrix may be challenging.

After reaching the marine environment, mainly due to accidental discharge or wastewater treatment plants’ effluents, NoV are highly resistant. Oysters farmed in coastal areas can accumulate NoV in their digestive tissues (DT) as they filter large amounts of water and thereby come into contact with numerous molecules and particles. We previously reported that some oyster species selectively accumulate NoV due to the presence of specific carbohydrates, similar to the human histo blood group antigens (HBGA and so called HBGA-like antigens) (Le Guyader et al., 2012). These carbohydrates, with a clear seasonal expression, favor for example GI.1 NoV bioaccumulation when compared to genotypes that are more often detected in the human population, like GII.3 or GII.4 (Maalouf et al., 2011; Yu et al., 2015). When oysters contaminated with multiple strains are consumed, there is a risk of disease for consumers, but it may also result in simultaneous single cell infections leading to recombination events (Le Guyader et al., 2008). These recombination events are mainly happening at the junction between ORF1 and ORF2 (van Beek et al., 2018). Therefore, understanding the full NoV diversity in oysters is important.

During the last years, next generation sequencing has increasingly been used to study microbial populations in environmental samples, allowing the detection of several cultured and non-cultured microorganisms (Nooij et al., 2018; Osunmakinde et al., 2018). Nevertheless virus detection in environmental samples is challenging in metagenomics studies due to the high levels of (background) host and microbial DNA, high virus diversity in sewage, and the high rate of, potentially novel, unclassifiable sequences (Bibby and Peccia, 2013; Hjelmsø et al., 2017; Nooij et al., 2018). In addition, low concentrations of NoV, the persistence of potential inhibitors that may prevent certain enzymatic reactions and the large diversity of other microorganisms present in the sample complicates the detection of NoV using metagenomic sequencing (Hata et al., 2017; Adriaenssens et al., 2018; Flaviani et al., 2018; Osunmakinde et al., 2018; Strubbia et al., 2019). Therefore, sequencing outcome is dependent on sample preparation, meaning that the relationship between sample concentration and the number of NoV reads may vary according to the method applied to concentrate and purify the nucleic acids (Hjelmsø et al., 2017; Fernandez-Cassi et al., 2018; Oechslin et al., 2018). A metabarcoding approach, which is amplicon based and thus requires specific primers, was proposed to sequence NoV from environmental samples allowing deep-sequencing even in samples with low virus concentrations and independently from the host background (Kazama et al., 2017; Oechslin et al., 2018). However, this approach and the design of the primers is limited to our current knowledge about NoV diversity and therefore will potentially miss the detection of new NoV strains that are genetically distinct from known diversity. Due to this limitation, metagenomic sequencing would be more suitable for the identification of new NoV strains (Fumian et al., 2019).

Here, we describe the performance of different concentration methods on the recovery of NoV from sewage and oyster samples. These methods allowed us to describe the NoV diversity in sewage samples and in oysters exposed to these sewages, to identify long sequences (>500 bp up to full genome) needed for strain identification, and to compare the NoV diversity in both sample types.

## Materials and Methods

### Samples Collection

Composite raw sewage sampled over 24-h were collected from a sewage treatment plant in a large city in western France (303 800 inhabitants). Three samples, which constituted of at least 5-L, were collected within 2 months (February and March 2018) and transported at 4°C to the laboratory, analyzed for the presence of NoV and kept frozen at −20°C in aliquots of 1 L.

Oysters (*Crassostrea gigas*) were directly purchased from the same producer few days before each experiment, analyzed for NoV contamination and kept at 4°C until use for bioaccumulation experiments.

### Bioaccumulation

Three bioaccumulation experiments (B1, B2, B3) were performed, one in February and two in March to limit oyster physiological variability and seawater composition. Aquariums were filled with 22-L of natural seawater seeded with adjusted volume of sewage to reach a comparable NoV concentration for all three experiences (around 10^7^ RNA copies/L). Then, 140 oysters were immersed for 24 h at 12 ± 1°C, under oxygenation.

### Sewage Preparation

Wastewater samples were prepared according to two different methods.

- For the polyethylene glycol (PEG) method, 40 mL of sample was adjusted to pH 4 by adding HCl, and adjusted to conductivity reading of 2000 μS by adding NaCl 5 M. After 5 min, 10 mL of 50% PEG 6000 solution (Sigma-Aldrich, St-Quentin France) was added. The mixture was incubated under gentle agitation overnight at 4°C and centrifuged at 13,500 × *g* for 90 min. The pellet was resuspended in 2 mL of glycine buffer 0.05 M (pH 9) and mixed with 2 mL of Chloroform-Butanol (vol/vol), vortexed at high speed for 30 s and centrifuged for 5 min at 11,000 × *g*. The aqueous phase was recovered and used for nucleic acid extraction.

- For the Pyro-PEG method, 4 mL of 10 mM sodium pyrophosphate decahydrate was added to 40 mL of sample and incubated for 40 min at room temperature under gentle agitation (Bisseux et al., 2018). Then, the mixture was sonicated for 1 min at maximum power in a cup-horn adaptor (Bandelin, HD 2200), followed by 1 min recovery on ice, which was repeated three times. After centrifugation for 20 min at 8,000 × *g*, supernatants were recovered, the pH adjusted to 4 and the conductivity was adjusted of 2000 μs by adding NaCl 5 M. After 5 min, 10 mL of 50% PEG 6000 solution (Sigma-Aldrich, St-Quentin France) was added and rocked for 1 h at 4°C. After centrifugation for 90 min at 13,500 × *g*, the pellet was resuspended in 2 mL of glycine buffer pH 9.

For both methods, the resuspended pellets were filtrated using a cascade of 5, 1.2, and 0.45 μm filter pores (Minisart NML 17594, NML17593, PES16533, and PES16532). The recovered filtrates were incubated for 1 h at 37°C with 2000 Units of OmniCleave Endonuclease^TM^ (Lucigen Corporation) and 100 μL of MgCl_2_ (100 mM).

### Oyster Preparation

Following bioaccumulation, oysters were immediately collected and shucked using a sterile knife. Flesh weight was recorded to calculate the allometric coefficient (flesh weight divided by DT weight), used to describe physiology of the animals (Polo et al., 2018). This coefficient was similar (14 to 10) for the three batches of oysters used for the experiments, presuming that they have similar filtering and physiology activity and are thus comparable for the three different experiments. The digestive tissues (DT) were dissected, chopped finely to homogenize, pooled, and distributed into 2 g aliquots, immediately frozen at −80°C.

Viruses were recovered using two methods. For both methods, 2 g of DT were incubated with 2 ml of proteinase K solution (30 U/mg, Sigma-Aldrich, France) for 15 min at 37°C and additional 15 min at 60°C. The mixtures were sonicated for 1 min at maximum power, followed by 1 min on ice, repeated three times. Supernatants were collected after centrifugation for 5 min at 3000 × *g* and kept at 4°C (method PK). For method PK-PEG, DT were treated using same conditions but then the supernatant was mixed with two volumes of PEG-NaCl 1.2 M and incubated under gentle agitation for 1 h at 4°C, before centrifugation at 11,000 × *g* for 20 min. Pellets were resuspended in 1 mL of glycine buffer (0.05 M) pH 9. Samples from both methods (PK and PK-PEG), were then incubated for 1 h at 37°C with 2000 Units of OmniCleave Endonuclease (Lucigen Corporation) and 100 μL of MgCl_2_ (100 mM).

### RNA Extraction and Purification

Nucleic acids extraction was performed using lysis buffer (bioMérieux, France) and the NucliSens kit (bioMérieux) followed by DNase treatment for 30 min at 37°C with 25 U TURBO^TM^ DNase (Ambion, Thermo Fisher Scientific, France). An additional RNA purification was performed using the RNA Clean & Concentrator^TM^-5 kit (Zymo Research, Irvine, CA, United States). Each sample was extracted three times and extracts were pooled to obtain a final volume of 300 μL. RNA extracts were stored at −80°C in aliquots of 30 μL.

### NoV Quantification in Sewage and DT

Noroviruses quantification was performed with a one-step digital RT-PCR using primers and probes targeting the conserved region at the beginning of ORF2 (Maalouf et al., 2011; Polo et al., 2016). Positive and negative controls were included in each series to validate the distribution of positive and negative wells. Quantification was done using the Poisson distribution (QuantStudio^TM^ 3D Analysis Suite^TM^ Cloud Software, version 3.0.3; Thermo Fisher Scientific, France) and the final result was expressed as RNA copies/μl (Polo et al., 2016).

### Library Preparation for Agnostic Metagenomic Sequencing

RNA extracts were transcribed into cDNA using Superscript II (Invitrogen, France) and non-ribosomal hexamers (Endoh et al., 2005). Libraries were prepared using the NEB Next Ultra DNA Library Prep Kit (New England BioLabs, France) according to the manufacturer’s instructions. Sequencing was performed using Illumina MiSeq technologies to generate 2 × 150 bp reads. All samples were sequenced agnostically (referred to as direct sequencing in the text). Each run corresponded to one bioaccumulation experiment (B1, B2 or B3) including the sewage sample (ww1, ww2 or ww3) prepared with the two methods and the DT (DT-1, DT-2, and DT-3) prepared with the two methods. Each run also included the reference sample (called Ref1, Ref2, and Ref3 according to the run) that correspond to the ww3 sample (extracted with the Pyro-PEG method) split after library preparation and sequenced in each run to compare the performance of the different sequencing runs.

### VirCapSeq-VERT Capture

Oysters’ DT were also analyzed with a targeted deep sequencing using VirCapSeq-VERT for viral enrichment (Wylie et al., 2015). In short, reverse transcription was performed using random hexamers and SuperScript III (Thermo Fisher Scientific) after which dsDNA synthesis was performed using Klenow (New England Biolabs, France). Libraries were prepared using the KAPA HyperPlus Kit (Roche, France) according to the manufacturer’s instructions with slight modifications. The shearing time was reduced to 3 min and adapters were diluted 1:10. After the adapter ligation an addition AMPure bead step was performed. The libraries were quantified and pooled equimolarly after which the capture experiment was performed (Wylie et al., 2015). Sequencing was performed on an Illumina MiSeq using the MiSeq Reagent KIT v3 (Illumina) to generate 2 × 300 bp reads.

### Sequence Analysis

Illumina adapters were removed from the raw short reads and resulting reads were trimmed from 3′ end to reach an average the Phred score ≥35 using QUASR (Watson et al., 2013). Resulting reads were *de novo* assembled using SPAdes v3.12.0 and family-specific viral contiguous sequences (call contigs) were classified and sorted by SLIM (Bankevich et al., 2012; Cotten et al., 2014). Then, SLIM outputs were filtered to select contigs with an identity score ≥85% and a minimum length of 500 bp to avoid too short and non-informative fragments. Partial overlapping contigs were further assembled into genomes using Geneious^®^ v. 11.1.5 and all SNPs (single nucleotide polymorphisms) between contigs were resolved by counting motifs in the raw read sets. For NoV, final contigs were genotyped using the online norovirus genotyping tool v2.0 (Kroneman et al., 2013). In order to estimate the read coverage, raw reads were mapped to the resulting contigs using Bowtie2 (v2.3.0) (Langmead and Salzberg, 2012).

Maximum likelihood trees were inferred with PhyML v3.0, using the general time reversible (GTR) nucleotide substitution model (Guindon et al., 2010). VP1 sequences representing different GI and GII genotypes were included in the phylogenetic trees. The GI and GII trees were inferred using an alignment of approximately 800 bp from the middle and end of VP1, respectively. Different regions were selected to be able to include the maximum amount of Illumina sequences.

## Results

This study aimed to optimize NoV recovery from sewage and oyster DT samples, to analyze subsequent reads obtained and then to finally compare strains detected in sewage samples and oyster samples contaminated with these sewages.

### NoV Concentrations in Sewage and Oyster Samples

For sewage samples, the Pyro-PEG method increased the recovery of NoV when compared to the PEG method, both in terms of concentrations (RNA copies per μl measured by dRT-PCR) and read numbers after NGS (Table 1). For the sewage sample (ww2), used for the second bioaccumulation experiment (B2), the NoV GI concentration was under the detection limit when treated with PEG, but increased up to more than 500 copies/mL with the pyro-PEG method. When comparing geometric means of the concentrations calculated for the three bioaccumulation experiments the number of NoV GI particles increased over 2 logs while the number of NoV GII particles increased 1 log when using the Pyro-PEG compared to only PEG.

**TABLE 1:** Data obtained from the three bioaccumulation experiments.

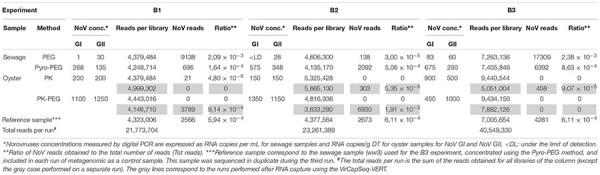

For oyster DT samples, NoV concentrations were always higher using the PK-PEG method, and the increase differed by genogroup (from 0.5 to 9 fold for NoV GI and from 2 to 7.6 fold for NoV GII). These concentrations were representative of oyster contaminations observed in highly polluted area.

### Reference Samples Sequencing

**FIGURE 1:**
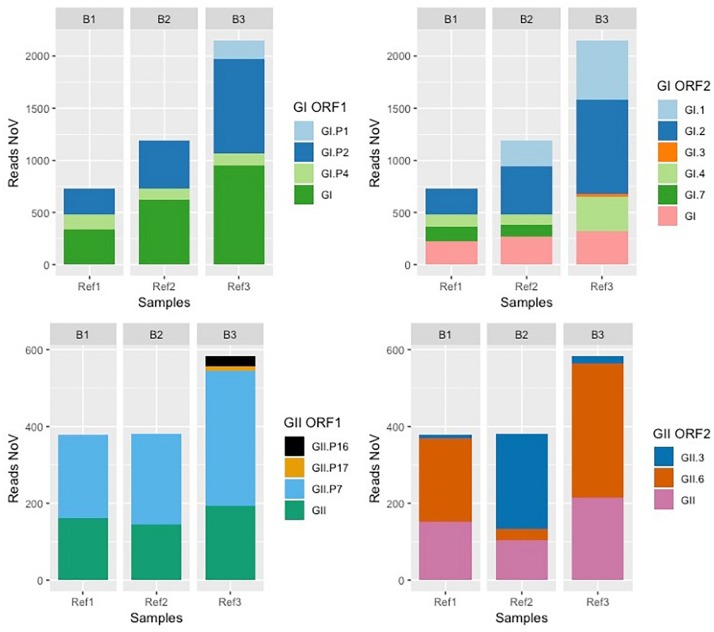
Bar plot reporting the reference sample sequenced during each bioaccumulation experiment. The sewage sample 3 used as a reference sample was included in each sequencing run performed for each experiment (B1, B2, and B3). Results obtained for this reference sample (Ref1, Ref2, and Ref3) for each run are reported as number of reads obtained for NoV GI (top two graphs) and NoV GII (bottom two graphs) corresponding to the polymerase (ORF1 on the left) and capsid protein (ORF2 on the right).

Each bioaccumulation experiment (B1, B2, and B3) was sequenced in separate sequence runs and the library of the sewage sample used for the third bioaccumulation (B3) was included in each run so it can be used as reference sample. The first and second run gave comparable numbers of total reads, while the third run produced two times more reads (Table 1). However, a comparable ratio of NoV reads were obtained from the reference sample in the three different sequence runs: in the first run 5,94 × 10^–4^ NoV reads per million sequence reads (rpm) were obtained and in the second and third run 6,11 × 10^–4^ NoV rpm were obtained. The diversity of NoV genotypes identified in the reference sample in the three different runs is comparable, with a higher number of NoV reads for each genotype? in the third run (Figure 1). Additionally, the third run of the reference sample (Ref3) yielded reads of NoV GI.3 and GII.P16 that were not detected in runs 1 and 2 (Figure 1). This confirms the importance to get sufficient sequence reads to be able to detect all strains that might be present.

### Method Comparison for NoV Sequencing From Sewage Samples

**FIGURE 2:**
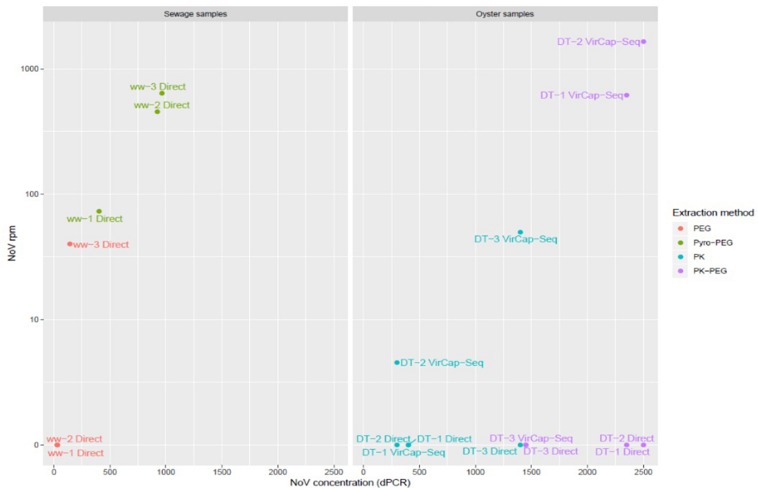
Scatter plot showing NoV concentrations and number of NoV reads. NoV concentrations expressed in RNAc/g of DT for oyster samples, and RNAc/mL in sewage samples (*x* axis), were plotted against the number of reads per million (rpm) (*y* axis). The legend on the right assign a color according the extraction methods: PK in red and PK-PEG in green for oyster samples (left table), and PEG in blue and Pyro-PEG in purple for sewage samples. The deep sequencing approach is specified for each sample: “Direct” for an agnostic approach or “VirCapSeq-VERT” when based on the capture experiment.

To evaluate the depth of NoV sequencing, we tested two different extractions protocols. We investigated if there was a relationship between the NoV concentration (sum of NoV GI and GII) and the number of NoV reads after NGS (Figure 2). Sewage samples treated with the Pyro-PEG method showed higher numbers of NoV reads in agreement with higher NoV concentrations obtained using this method. A small number of reads of NoV GIV were obtained from samples treated with Pyro-PEG method but not from those treated with the PEG method.

**FIGURE 3:**
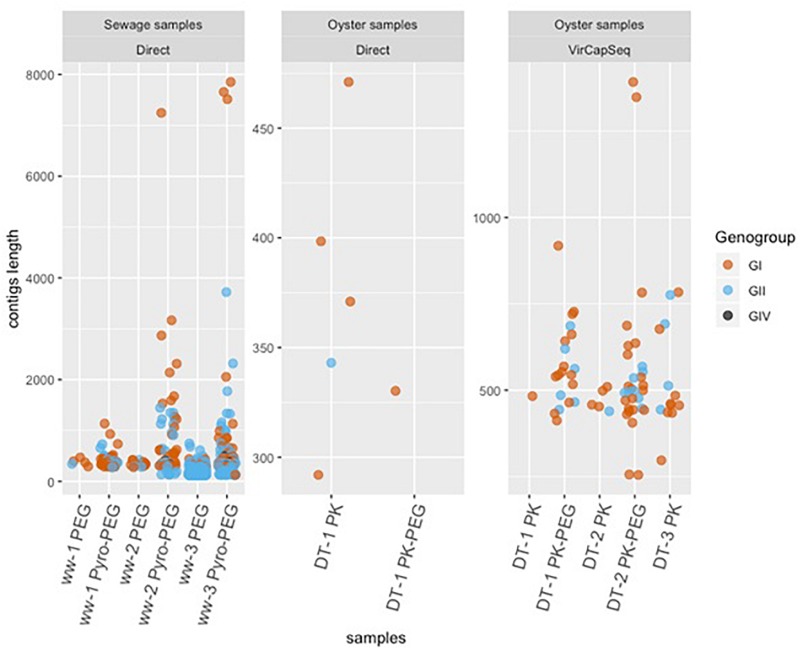
Scatter plot of jittered data showing the length of contigs. NoV GI (brown), GII (blue), and GIV (black) in oysters (DT) and wastewater (ww) samples are reported with a scale proportional to the longest contigs found in each sample type.

Another aim of this method comparison was to increase NoV contig length in order to increase the identification and classification of NoV strains present in our samples. For sewage samples, only the Pyro-PEG method resulted in contigs with a length over 1000 nt for both genogroups and even up to 7000 nt (full genomes) for NoV GI (Figure 3). Using the Pyro-PEG methods, six full NoV GI genomes were recovered from sewage samples (Table 2). For NoV GII, the longest sequence detected was a GII.P7-GII.6 (5050 nt). For some other strains (nearly) full genomes were not obtained but the overlapping region of ORF1 and ORF2 region was sequenced allowing for dual genotyping and classification (Table 3). Some sequences such as GII.6, and GII.4 were detected in all three sewage samples that is not surprising as samples were collected from the same sewage treatment plant over a short period of time. Quite a large diversity of GI strains was also identified in ww-2 and ww-3, confirming the interest of sewage samples to identify some strains that may not been seen in clinical cases.

**TABLE 2:** Complete NoV genomes identified.

**Sample (run)**	**Sample code**	**ORF 1**	**ORF 2**	**Length**	**BLAST score^∗^**	**Ref. sequence**^#^	**Identity**^#^
Ref sample (1)	G19_001	GI.P1	GI.1	7134	81.54	KF306212.1	87%
Ref sample (2)	G19_004	GI.P2	GI.2	7619	92.27	KF306212.1	99%
ww-3 (3)	G19_005	GI.P4	GI.4	7581	91.10	LN854563.1	96%
ww-3 (3)	G19_006	GI.P2	GI.2	7433	94.56	KF306212.1	99%
Ref sample (2)	G19_002	GI.P1	GI.1	7173	84.99	NC_001959.2	85%
ww-3 (3)	G19_003	GI.P1	GI.1	7363	84.72	NC_001959.2	85%

*Blast score (^∗^) was assigned using the NoV typing tool v2.0 (Kroneman et al., 2011), and reference sequence and percentage of identity were obtained using BLAST (NCBI) (^#^).*

**TABLE 3:** Noroviruses GII sequences for which it was possible to identify both ORFs (ORF1 and ORF2).

**Sample (run)**	**Sample code**	**ORF 1**	**ORF 2**	**Length**	**BLAST score^∗^**	**Ref. sequence**^#^	**Identity**^#^
ww-3 (3)	G19_007	GII.P7	GII.6	3720	85.63	MH218642.1	98%
Ref sample (3)	G19_008	GII.P7	GII.6	5050	87.11	MH218642.1	98%
Ref sample (1)	G19_009	GII.P7	GII.6	4292	86.55	MH218642.1	98%
Ref sample (2)	G19_011	GII.P17	GII.17	1374	81.57	KU561249.1	98%
TD (1)	G19_012	GII.P16	GII.4 Syd 2012	686	89.85	KY210980.1	99%

*Reference sequence and percentage identity were obtained using BLAST (NCBI). Blast score (^∗^) was assigned using the NoV typing tool v2.0 (Kroneman et al., 2011), and reference sequence and percentage of identity were obtained using BLAST (NCBI) (^#^).*

### Method Comparison for NoV Sequencing From Oyster Samples

For DT samples, only 21 NoV reads were obtained without enrichment and all of them were from the sample DT-1 treated with PK (Table 1). The preparation of the sequence libraries were repeated using a specific enrichment for vertebrate viruses (VirCapSeq-VERT). This sequence run resulted in 31,377,552 sequence reads and NoV contigs were recovered from all three DT (DT-1, DT-2, and DT-3). A positive trend between the number of reads and NoV concentrations was observed after using VirCapSeq-VERT. The PK-PEG method provided a higher amount of NoV reads for DT-1 and DT-2, with 3789 and 6930 NoV reads, respectively, but not for DT-3. Using the PK method, 303 and 458 NoV reads were obtained from DT-2 and DT-3, but none from DT-1.

**FIGURE 4:**
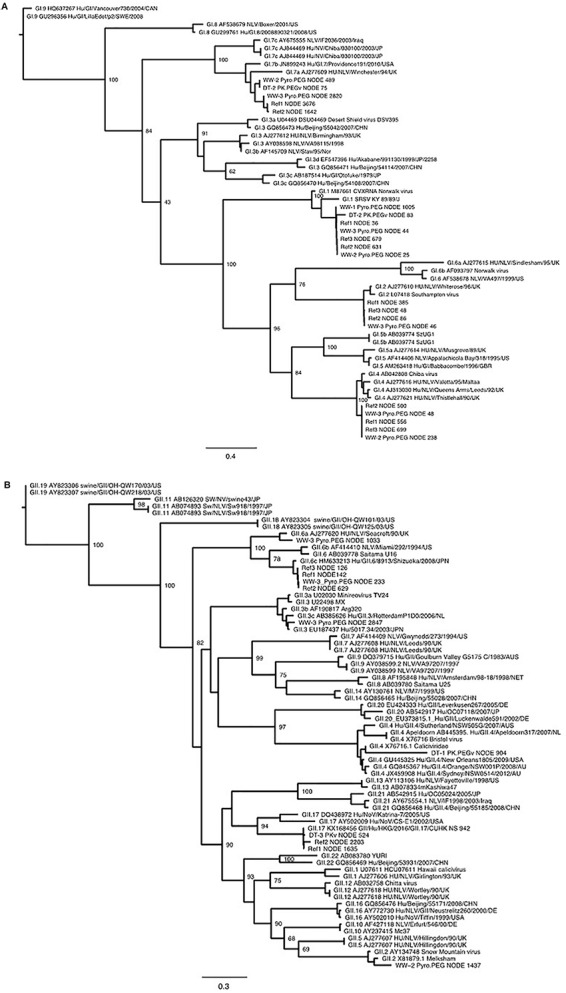
Phylogenetic trees inferred from partial GI **(A)** and GII **(B)** VP1 sequences using PhyML. All identified sequences in sewage sample (ww-1, ww-2, and ww-3), the reference sample (Ref1, Ref2, and Ref 3) and the oyster Digestive tissues (DT-1, DT-2, and DT-3). The method used to prepare the nucleic acids are reported, except for the Ref sample as the library was prepared only after the Pyro-PEG method.

Direct sequencing from oysters’ DT did not provide enough NoV reads to allow strain identification. Using VirCapSeq-VERT, the PK-PEG method provided the longest contig length and in total 57 contigs of >500 nt length were obtained, compared to 19 using the PK method. The longest consensus sequence obtained from oysters’ samples was 1393 nt and allowed the identification of a GI.P7-GI.7 strain. The diversity of sequences detected is quite large both for NoV GI and GII (Figures 4A,B).

### Comparing NoV Diversity in Sewage and in Bioaccumulated Oysters

**FIGURE 5:**
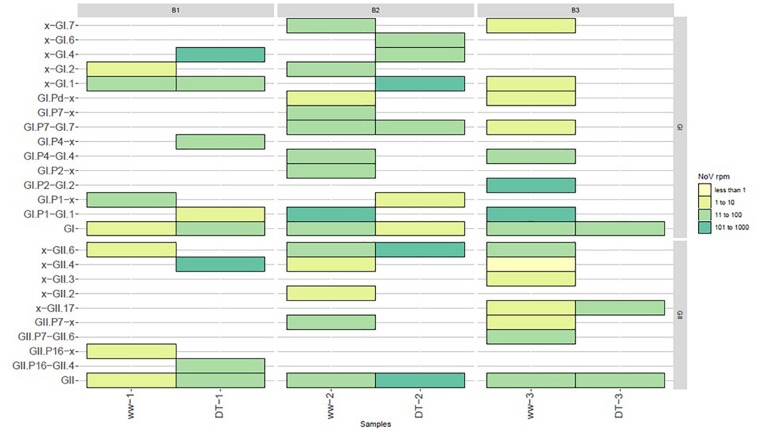
Diversity of NoV strains reported in sewage (ww) and oysters (DT) samples. Values were grouped into four categories and depicted with the following colors: light yellow for values between ≥1 and ≤10 reads, lime between ≥11 and ≤100, light green between ≥101 and ≤1000, dark green between ≥1001 and ≤10000.

One objective of this work was to compare the diversity of NoV in sewage samples and in oysters contaminated with these sewage samples. Due to the failure to obtain NoV reads after direct sequencing, this led us to use VirCapSeq-VERT sequencing. Different methods may induce differences in strains recovery or identifications nevertheless our results allowed us to make some observations (Figure 5). Furthermore, identical genotypes were detected in sewage and DT for GI (GI.p1-GI.1, GI.p4-GI.4, and GI.p7-G1.p7 strains, Figure 4A) and for GII (GII.6 and GII.P16-GII.4, Figure 4B). Phylogenetic trees inferred for partial VP1 GI GII sequences (Figures 4A,B) showed that these sequences were identical. Unfortunately, the third experiment provides only few sequences from DT (one GI that could not be typed and one GII.17 strain).

### Diversity of Other Viruses in Environmental Samples

**FIGURE 6:**
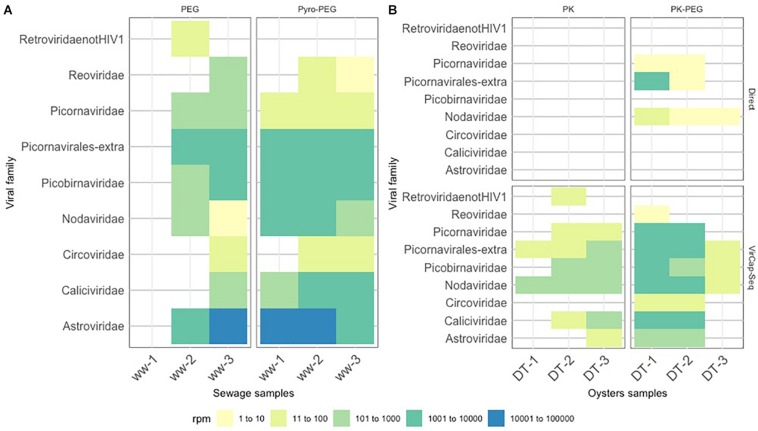
Heat-map representing virus diversity and the number of reads in each sample. Reported contigs with a sequence length ≥500 bp and identity score ≥85% were mapped toward the contigs obtained with SLIM. Values were grouped into five categories and depicted with the following colors: light yellow for values between ≥1 and ≤10 reads, lime between ≥11 and ≤100, light green between ≥101 and ≤1000, dark green between ≥1001 and ≤10000 and blue between ≥10001 and ≤100000 reads. **(A)** displays the results of the sewage samples (ww) used in the three bioaccumulation experiments, while **(B)** displays the results of the oyster digestive tissues (DT).

Among the eukaryotic viruses identified, a number of assembled contigs were shown to belong to other human enteric virus families including members of the *Reoviridae*, *Picornaviridae*, *Caliciviridae*, and *Astroviridae*. For sewage samples, the Pyro-PEG method provided a higher number and diversity of viral reads for all samples (Figure 6A). This is especially true for sewage sample 1 (ww-1) for which no read corresponding to human enteric viruses was identified using the PEG method while using Pyro-PEG allowed the identification of several different viruses. Members of the *Astroviridae* family were most abundantly present in sewage samples using both methods and four complete genomes were identified (one type 1, two type 2, and one type 3). Only in one occasion (ww-3), reads of the *Reoviridae* family were 10 time more represented after the PEG method compared to the pyro-PEG method, but contigs did not allowed strain identification. Concerning viruses belonging to the *Caliciviridae* family, a consistent improvement in sequence read recovery and contig length was reported with Pyro-PEG for all three sewage samples (between 1,000 and 10,000 times more), in agreement with the NoV concentrations. Three sapovirus complete genomes were obtained (GI.2, GI.3, and GII.1). For DT samples (DT-1, DT-2, and DT-3), very few sequences were identified after direct sequencing and only some after the PK-PEG method. This difference between the two protocols persisted after applying the VirCapSeq-VERT capture. The number of viral reads obtained was much higher for all viral families in samples treated with PK-PEG method, except for DT-3 for which very few reads were obtained (Figure 6B). Furthermore, the PK-PEG method led to the identification of viruses belonging to the *Picornaviridae*, *Picobirnaviridae*, *Caliciviridae*, and *Astroviridae* families. Comparing viral families detected both in sewage and DT, only sequence reads derived from the *Reoviridae* family were reported in ww- 2 and 3 (but with read numbers <100), while they could not be detected in the corresponding DT extracts, suggesting a low accumulation of rotavirus by oysters.

## Discussion

There are only a limited number of protocols published for metagenomic NoV sequencing directly from sewage or oyster samples. Here, we evaluated different methods to improve the characterization of human enteric viruses, with a special focus on NoV, in sewage samples and oysters contaminated with these sewage samples using next generation sequencing. Our final goal was to obtain full NoV genomes to be able to study NoV diversity in environmental samples.

Based on experience in sewage analysis using PEG, we optimized the elution/concentration step to increase virus recovery (da Silva et al., 2007). One protocol is based on acidification to enhance binding of the viral capsid to particles according to their isoelectric point before PEG precipitation, a method found efficient to detect low levels of NoV in oysters (Shieh et al., 1999). The other method includes first a chemical elution step using sodium pyrophosphate combined with a sonication step, before the PEG concentration (Bisseux et al., 2018). This sonication step favors the disruption of agglomerates, enhances viral particles elution from organic fragments and reduce the amount of bacteria (Ettayebi et al., 2016; Santiana et al., 2018). This Pyro-PEG method was found efficient as it allowed the recovery of the highest number of NoV reads and was reproducible, as demonstrated by the increased proportion of NoV reads yielded with this approach when compared to the PEG method.

Using the same rationale, the sonication step was also applied to oyster DT after enzymatic elution of viral particles, before nuclease treatment or before PEG precipitation (Atmar et al., 1995). The impact of some added purification steps may not increase NoV concentrations, but by the elimination of background nucleic acids, they may increase the ratio of NoV reads and the quality of contigs obtained after NGS. In our hands, the PEG approach was already found valuable for stool or sewage samples by allowing longer NoV contig recovery and thus better genotyping (Strubbia et al., 2019). PEG precipitation has also been found useful for the recovery of spiked murine norovirus and human adenovirus, and for NoV metabarcoding, while a combination of centrifugation, filtration and chloroform treatment was found efficient to analyze the virome of environmental samples (Bibby and Peccia, 2013; Hjelmsø et al., 2017; Kazama et al., 2017). Indeed different sample types may need adapted approaches to optimize virus recovery since for instance non-specific approaches such as cryo-fragmentation or mechanical mikro-dismembrator on food samples failed to detect NoV in strawberries (Wylezich et al., 2018). The finding of two NoV reads out of 28,856,294 obtained sequence reads from berries implicated in an outbreak and positive for NoV according to the NoV detection ISO method, showed that improvement is needed (Bartsch et al., 2018).

Since the study is based on the comparison of three individual sequence runs, we used one sewage extract as reference sample to control for variability in deep sequencing. We found this approach valuable as it allowed us to verify that all runs provide similar results. As different aliquots of the library were used, we cannot expect to identified exactly the same sequences, but the global sequence distributions were comparable. This approach also showed that more reads allowed the identification of more strains, an important point when dealing with environmental samples with low contamination levels. Indeed, we found a correlation between the NoV concentrations and the number of reads in sewage samples. This was also verified with the oyster DT extracts as NoV concentrations obtained were too low for an agnostic approach, even if the PEG step after the PK enzymatic elution of the virus increased NoV recovery. For these samples, only the use of VirCapSeq-VERT assay yielded enough NoV reads to allow sequence assembly and strain identification. This suggest that our protocols were able to recover NoV particles but more optimizations are needed to improve the recovery of NoV sequences and probably also for other human enteric viruses. The need to enrich for viral sequences was also needed in some clinical samples confirming the difficulty for viral sequencing (Wylie et al., 2018).

One of the goals of this study was to describe NoV diversity in sewage samples and in artificially contaminated oyster samples. Several NoV strains were identified both in sewage and oyster samples (such as GI.1, GI.4, and GI.7), while some GI.1 was only detected in DT. Few years ago, we demonstrated that oysters are not passive filters but they can actively select some strains, possible explanation for some greater implication of NoV GI strains in oyster-related outbreaks compared to other ways of transmission (Le Guyader et al., 2012; Verhoef et al., 2015; Yu et al., 2015). To fully understand the role played by ligands it is important to have methods describing the viral diversity in oysters, including the presence of other human enteric viruses. Regarding NoV, it was surprising to find only few sequences of NoV GII.4 in sewage as it is known to be the dominant cause of NoV outbreaks and thus abundant in the environment especially during winter season (de Graaf et al., 2016). This is unlikely to be due to a bias induced by the protocol used, as the PEG method has already be found efficient to characterize GII.4 NoV but further investigation has to be done (Kazama et al., 2017).

To date, no studies have been conducted using metagenomics analysis on contaminated oysters in relation to NoV contamination. Although the present work is based on artificially contaminated oysters, it represents a first step on developing methods to detect NoV from oysters’ DT using deep sequencing technologies. Further developments are still necessary to enrich for NoV particles during sample treatment and virus recovery as well as to reduce the host background in order to improve deep sequencing analysis and to obtain longer NoV sequences, necessary for accurate NoV classification. Despite low NoV concentrations in oysters, they have been implicated in some food borne outbreaks (Polo et al., 2016). Thus, developing sensitive methods for oyster analysis is important to clarify their role in NoV transmission. This work also brings further evidences of the role played by oysters on virus selection and thus will contribute to the understanding of the molecular epidemiology of norovirus. Such approach can be later extended to other food that represent a risk for consumers (Forbes et al., 2017; Cocolin et al., 2018).

## Data Availability Statement

The short-reads data for this study has been deposited in the European Nucleotide Archive (ENA) http://www.ebi.ac.uk/ena/dta/view/PRJEB34617 (ERS3781162–3781182). All full genome sequences were deposited in GenBank and are available under the accession numbers MK956173–MK956178 for NoV genogroup I, numbers MK956197–MK956200 for genogroup II, and numbers MN510436–MN510441 for astrovirus and sapovirus.

## Author Contributions

All authors contributed to the work either to design the study, to perform the experiments, to analyze the data, to discuss the results, and to write the manuscript.

## Conflict of Interest

The authors declare that the research was conducted in the absence of any commercial or financial relationships that could be construed as a potential conflict of interest.
